# Short-term real-world outcomes of diabetic macular edema treated with intravitreal faricimab

**DOI:** 10.1371/journal.pone.0323088

**Published:** 2025-05-13

**Authors:** Toshiaki Hirakata, Fumihiro Hara, Yuta Nochi, Daisuke Shinohara, Shutaro Yamamoto, Yoshimune Hiratsuka, Shintaro Nakao

**Affiliations:** Department of Ophthalmology, Juntendo University Faculty of Medicine, Tokyo, Japan; Akita University: Akita Daigaku, JAPAN

## Abstract

Faricimab, a new drug for diabetic macular edema (DME), was made available in Japan in 2022. However, few reports have been published about its treatment outcomes in clinical practice. To assess the short-term outcomes of intravitreal faricimab (IVF) in patients with DME, the records of patients with DME receiving IVF therapy between July 2022 and July 2023 at Juntendo University Hospital were retrospectively reviewed. Their best-corrected visual acuities in the logarithm of the minimum angle of resolution units and central macular thicknesses were compared at baseline and one month after the final IVF. Eighteen patients and 22 eyes were included and allocated to the naïve and switched groups: 12 cases (15 eyes) and 6 cases (7 eyes), respectively. The best-corrected visual acuity improved for the naïve group, but no difference was observed for the switched group. In contrast, the central macular thickness improved for both the naïve and switched groups. IVF demonstrated good short-term outcomes for DME, suggesting that it is effective for DME in clinical practice.

## Introduction

Diabetic retinopathy (DR) is the most common microvascular complication in patients with diabetes [1]. Diabetic macular edema (DME), which is a component of DR, causes vision loss. Intravitreal injections of anti-vascular endothelial growth factor (anti-VEGF) agents are currently the most effective treatment for DME [2]. However, they have several limitations, including the need for repetitive injections and insufficient response in a subset of patients.

Faricimab (Roche/Genentech, Basel, Switzerland) was developed as a bispecific antibody to bind VEGF-A and Ang2 [3]. It was approved in Japan in March 2022 and its use began in May 2022. It has anti-Ang2 effects and is expected to be more effective for DME than previous treatments [4]. It is also expected to be effective for cases of ineffectiveness or resistance to existing drugs such as ranibizumab and aflibercept. Indeed, a few previous report showed the efficacy of switching to faricimab in eyes with DME refractory to aflibercept [5–7].

YOSEMITE and RHINE were, global, multicenter, randomized, double-masked, active-comparator–controlled, phase 3 trials of faricimab in patients with DME, including Japanese patients [4,8]. However, while clinical trials such as YOSEMITE and RHINE have demonstrated the efficacy and safety of faricimab, real-world data is essential to validate these findings in diverse patient populations and clinical settings. Moreover, the number of initial injections during the introduction period was 4 or 6, and the interval between injections was fixed or treat and extend (TAE) per personalized treatment interval, in these randomized trials.

This study examines the short-term real-world outcomes of faricimab in DME, focusing on its application under pro re nata (PRN) or TAE regimens—approaches less explored in previous studies. This study aimed to determine the short-term real-world efficacy of faricimab for the treatment of DME in Japanese patients, including both PRN and TAE regimens.

## Materials and methods

This study included patients treated with intravitreal faricimab (IVF) for DME between First, July, 2022 and 31th, July, 2023. The exclusion criteria were cases that could not be followed up due to complications of diseases that cause retinal exudative changes other than DME during follow-up, and cases that self-interrupted their hospital visits. Patients with a history of previous vitrectomy were not excluded from this study. IVF was administered when retinal exudative changes (intraretinal and subretinal fluids) were detected by optical coherence tomography (OCT). The administration method of IVF was pro re nata (PRN) or treat and extend (TAE). The follow-up lasted from 1^st^, July, 2022–31th, August, 2023. Of the 15 cases treated with PRN, 3 cases received 3 monthly loading injections of faricimab, and 12 did not have an induction period. On the other hand, of the 7 cases treated with TAE, 5 cases received 3 monthly loading injection of faricimab and 2 cases did not. This study was conducted in accordance with the Declaration of Helsinki and approved by the Institutional Review Board of Juntendo University (16–292). Written informed consent from the patients enrolled in this study was waived based on the optout method facilitated through our hospital bulletin board. All patient data were anonymized and collected retrospectively. The data were accessed and analyzed from 1^st^, October, 2023–31th, December, 2023.

The primary outcome of the study is the change in visual acuity from baseline. Secondary outcome was the change of central macular thickness (CMT) in OCT from baseline. All patients underwent comprehensive ophthalmic examinations, including logarithm of the minimum angle of resolution best-corrected visual acuity (logMAR BCVA), inter ocular pressure (IOP), fundus ophthalmoscopy, and spectral-domain OCT (SD-OCT; Carl Zeiss and Heidelberg). OCT analyzed the central macular thickness (CMT). OCT data for CMT was collected using the Cirrus HD-OCT 512 × 128 macular cube protocol, or Heidelberg SD-OCT volume scan protocol. The baseline data obtained immediately before IVF administration and data from one month after the last IVF during the follow-up were compared. The statistical analyses were performed using paired T test in Prism 10 software. A p-value of < 0.05 was considered as statistically significant.

In order to confirm the safety of faricimab, Adverse events related to faricimab during the research period were also investigated. Aligning with the YOSEMITE and RHINE trials, endophthalmitis, retinal detachment, or systemic adverse events were checked.

## Results

This study included 18 patients with 23 consecutive eyes treated with intravitreal faricimab (IVF) for DME between First, July, 2022 and 31th, July, 2023. They were divided into the naïve (15 eyes of 12 patients) and switched (seven eyes of six patients) groups, and their medical records were retrospectively reviewed. All patients in switched group were inadequately effective cases, with no resolution of retinal fluid on OCT after their prior drug administration. For the switched cases, one patient was excluded because of concomitant age-related macular degeneration. Finally, 22 eyes from the 18 patients with DME treated with IVF were included in this study.

The clinical characteristics of the patients are summarized in Table 1. There were 12 male and 11 female, and mean age was 63 ± 15 years old. As for diabetes control, the mean HbA1c at baseline was 7.7 ± 1.2 in the naïve group, and 7.2 ± 0.9 in the switched group. Diabetic retinopathy stages in the naïve group were SDR in two eyes, PPDR in eight eyes, and PDR in five eyes; in the switch group were SDR in two eyes, and PPDR in five eyes. Sixteen of the 22 eyes had a history of panretinal photocoagulation. The types of DME were as follows: 5 eyes of cystoid macular edema (CME), 5 eyes of sponge and 8 eyes of CME + sponge, 1 case of CME + SRF, and 3 cases of sponge + SRF.

**Table 1:** Demographic all patients.

	Naïve	Switch	All case
**Case (eye)**	12 (15)	6 (7)	18 (22)
**Male (female)**	9 (2)	3 (4)	12 (6)
**HbA1c (%)**	7.7 ± 1.2	7.2 ± 0.9	7.5 ± 1.1
**DR stage (eye)**			
**SDR**	2	2	4
**PPDR**	8	5	13
**PDR**	5	0	5
**DME type (eye)**			
**CME**	5	0	5
**Sponge**	4	1	5
**SRF**	0	0	0
**CME + sponge**	4	4	8
**CME + SRF**	1	0	1
**Sponge + SRF**	1	2	3
**Past history of PRP (eye)**	11	5	16
**The number of previous anti-VEGF**		7.6 ± 9.2	
**Past history of vitrectomy (eye)**	2 (2)	0 (0)	2 (2)
**The number of IVF**	2.7 ± 1.3	4.2 ± 2.4	3.3 ± 1.9
**Treatment protocol of IVF**			
**PRN (eye)**	11	4	15
**Loading (+)**	1	2	3
**Loading (-)**	10	2	12
**TAE (eye)**	4	3	7
**Loading (+)**	2	3	5
**Loading (-)**	2	0	2
**Mean follow-up period (months)**	5.1 ± 3.7	8.4 ± 1.7	6.3 ± 3.6

CME, cystoid macular edema; DME, diabetic macular edema; DR, diabetic reritnopathy; IVF, intravitreal faricimab; PDR, proliferatiive diacitic retinopathy: PPDR, pre-proleferative diabetic retinoathy; PRN, pro re nata; PRP, panretinal photocoagulation; SDR, simple diabetic retinopathy; SRF, subretinal fluid; TAE, treat and extend; VEGF, vascular endotherial growth factor.

The mean follow-up duration was 6.3 ± 3.6 months. The number of intravitreal faricimab (IVFs) was 3.3 ± 1.9, 2.7 ± 1.3, and 4.2 ± 2.4 for the all cases and the naïve and switched groups, respectively. For the switched group, the number of previous anti-VEGF injections was 7.6 ± 9.2. Five aflibercept and two ranibizumab drugs were previously administered as anti-VEGF drugs before switching to faricimab. None of patients had a history of intravitreal steroid injections. Two eyes in the naive group had a history of vitrectomy for proliferative diabetic retinopathy.

**Fig 1:**
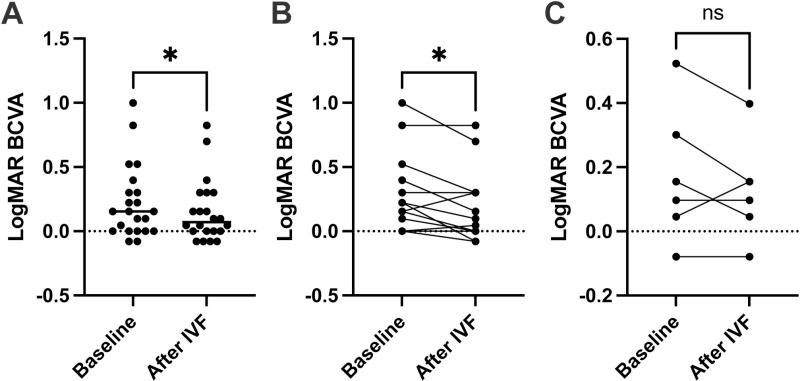
Visual acuity improved in the naïve group treated with IVF. **(A)** For all cases, the logarithm of the minimum angle of resolution best-corrected visual acuity (LogMAR BCVA) improved after intravitreal faricimab (IVF) administration. **(B)** For the naïve cases, the LogMAR BCVA also improved after IVF. **(C)** For the switched cases, no change in the LogMAR BCVA was observed. Data were analyzed using the paired t-test. *P < 0.05, naïve vs. after IVF.

The change in the logMAR BCVA is shown in Fig 1. At the baseline, the logMAR BCVA for the all cases, naïve and switched groups, was 0.21 ± 0.28, 0.24 ± 0.31, and 0.14 ± 0.20, respectively. At the endpoint, the logMAR BCVA for the all cases, naïve and switched groups, was 0.14 ± 0.24, 0.16 ± 0.26, and 0.08 ± 0.15, respectively. For all cases (p = 0.01) and the naïve group (p = 0.03), the logMAR BCVA significantly improved after IVF (Fig 1B). In contrast, the logMAR BCVA at baseline and after IVF were not different for the switched group (p = 0.31) (Fig 1C).

**Fig 2:**
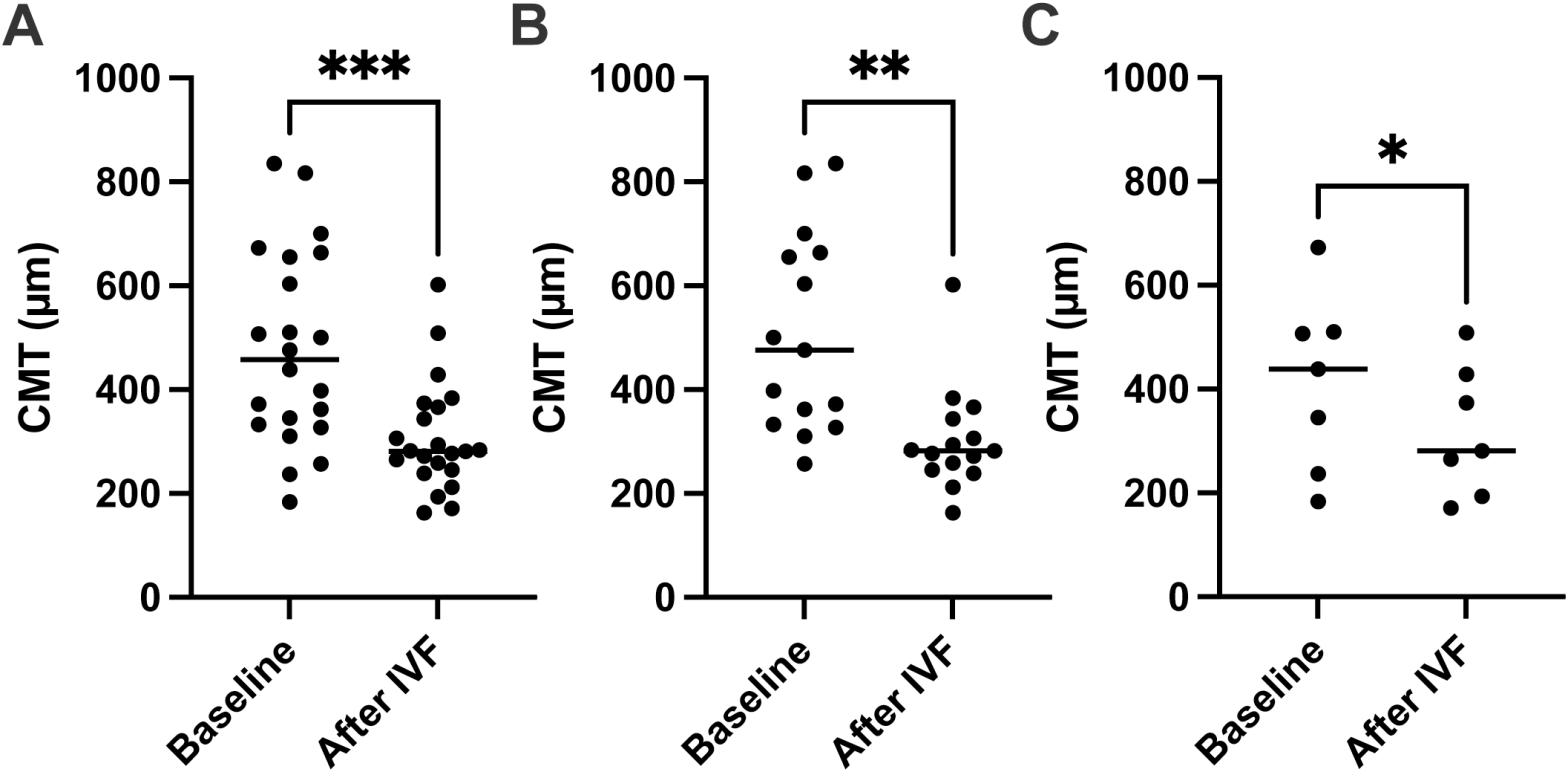
CMT improved for both the naïve and switched groups after IVF. **(A)** For all cases, central macular thickness (CMT) improved after intravitreal faricimab (IVF) administration. **(B)** CMT improved after IVF for the naïve cases. **(C)** CMT also improved after IVF for the switched cases. Data were analyzed using the paired t-test. *P < 0.05, **P < 0.01, ***P < 0.001, naïve vs. after IVF.

**Fig 3:**
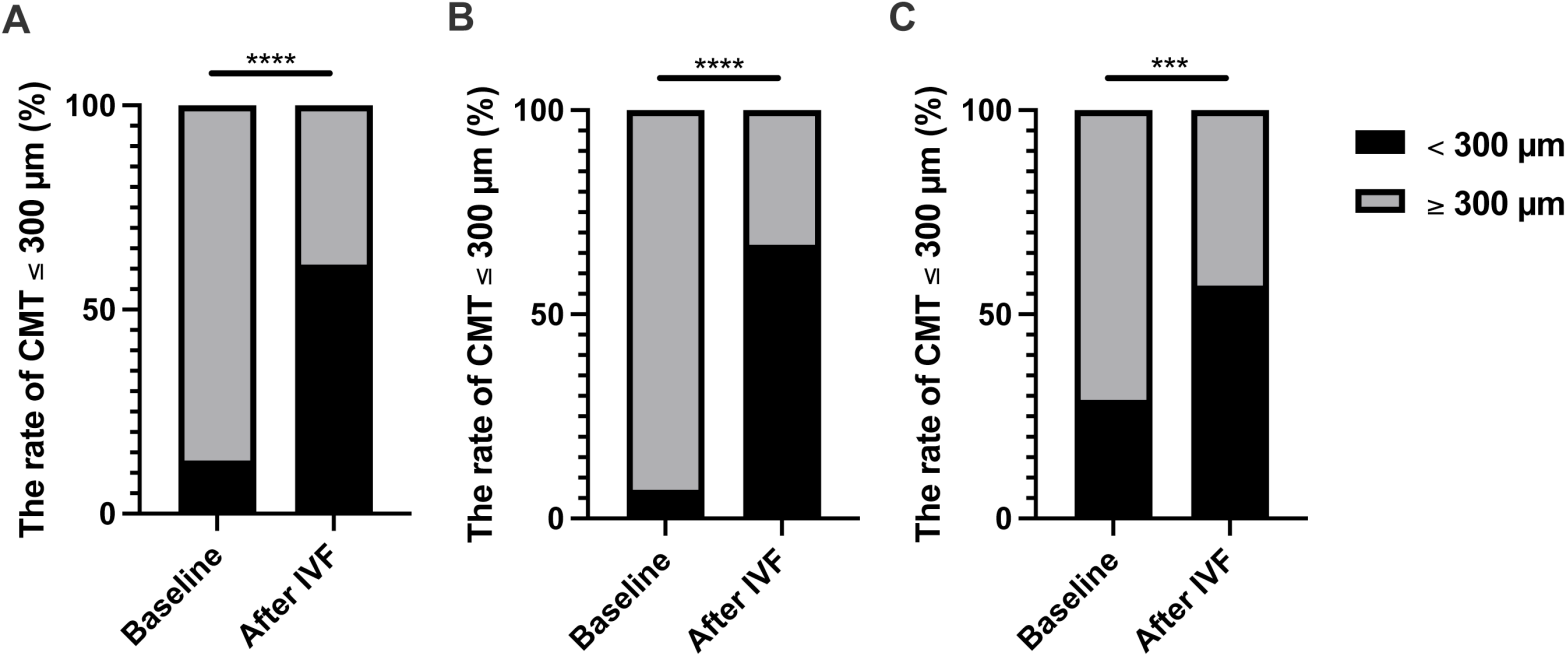
IVF treatment increased the proportion of CMTs below 300 µm. (A) For all cases, central macular thicknesses (CMT) of ≤ 300 µm improved after intravitreal faricimab (IVF). (B) For the naïve cases, CMT improved after IVF. (C) For the switched cases, CMT also improved after IVF. Data were analyzed using Fisher’s exact test. ***P < 0.001, ****P < 0.0001, naïve vs. after IVF.

Changes in CMTs are shown in Fig 2. At the baseline, CMT for the all cases, naïve and switched groups, was 478 ± 182 µm, 507 ± 185 µm, and 414 ± 158, respectively. At the endpoint, CMT for the all cases, naïve and switched groups, was 353 ± 152 µm, 359 ± 153 µm, and 341 ± 148 µm, respectively. The CMT significantly improved after IVF from the baseline for all cases (p < 0.001), naïve (p = 0.002) and switched (p = 0.04) groups (Figs 2A–C). Additionally, the percentage of patients with CMT of under 300 µm increased significantly in both the naïve and switched groups after IVF (Figs 3A–C).

No significant ocular or systemic adverse events were observed during the follow-up period. Both baseline IOP before IVF treatment (baseline) and final IOP (final visit) were in the normal range and did not differ in both naïve and switched groups.

## Discussion

In the present study, DME treatment with IVF was assessed retrospectively. IVF improved CMT for both the naïve and switched groups, suggesting a reduction in the retinal fluid. Visual acuity improved for the naïve but not for the switched group. These suggest that IVF is more effective at improving retinal morphological changes in DME. Our findings align with the established safety profile of faricimab, as no significant adverse events were observed.

In YOSEMITE and RHINE studies, enrolled patients were received intravitreal faricimab 6.0 mg every-8-week after 6 initial every-4-week doses, or intravitreal faricimab 6.0 mg per personalized treatment interval after a minimum of 4 initial every-4-week doses. [4,9]. In strictly controlled clinical studies, the number of injections tends to be higher than in actual clinical practice. In the randomized trials, four or six injections were used during the induction period, but in this study, the number of injections was 2.9 ± 1.8 in the PRN group and 4.6 ± 1.4 in the TAE group during the average follow-up period of 6.3 ± 3.6 months.

68% of the patients included in this study were treated with PRN. In addition, many patients did not have an induction period. This is different from previous randomized controlled trials conducted under strict protocols. Even so, the results of this study show that faricimab is effective. This suggests that faricimab may be effective in improving diabetic macular edema with fewer injections. Furthermore, it may also lead to a reduction in the financial burden on patients.

On the other hand, there were also many cases in this study that did not undergo the induction period. In the past, there have been reports that the prognosis for visual acuity is better with more injections during the induction period for anti-VEGF drugs [10], so the long-term prognosis for faricimab is an issue that needs to be examined in the future.

This was a retrospective study, and the methods of drug administration varied (PRN or TAE, with or without the administration period of IVF). Compared to the previous randomized trials [8], which included Japanese patients and demonstrated consistent BCVA and CMT improvements under standardized protocols, our real-world study showed variability, particularly in the switched group. This difference highlights the challenges in achieving uniform outcomes outside controlled environments. Cases in which no induction phase was provided were included, and the reasons for this were recognized, including the financial burden of the patient and fear of injecting the drug into the eye. Cases for which the exact number of injections required was not administered were also included, suggesting that this study reflects the actual clinical real-world aspects of the study.

This drug, anti-Ang2, has a new effect and is expected to be more effective for DME than previous treatments [3]. It is also expected to be effective for cases of ineffectiveness or resistance to existing drugs such as ranibizumab and aflibercept. In this study, naïve patients showed improvements in visual acuity and retinal thickness, suggesting good fluid control. In contrast, the switched case group showed improvement in retinal thickness but not in visual acuity.

The risk factors for poor visual improvement after DME treatment for the naïve group were older age and poor vision before treatment [11]. Murakami et al. showed that visual acuity was significantly worse for patients with cystoid macular edema than for those with the serous retinal detachment or diffuse type [12]. Moreover, a disrupted ELM or parafoveal thickening was significantly correlated with poor visual acuity of patients with diffuse DME. However, the switched group in this study showed no obvious abnormalities in the outer retinal layers on OCT. Some previous reports have shown the efficacy of IVF for the switched cases [13–15]. These differences in results may be attributed to the small sample in this study, given the trend toward improvement in this study. Additionally, in the switch group, the baseline decimal visual acuity of 5 out of 8 eyes was greater than (0.7). This suggests that there may have been a ceiling effect as to why there was no significant difference in visual acuity improvement. The pathomorphology and photoreceptor statuses at the fovea and retinal edema in the parafovea should be continually considered as prognostic factors for DME.

This study has several limitations. First, the sample was small. Second, the IVF treatment methods varied from case to case. Third, the follow-up was relatively short. We intend to continue this longitudinal study using additional cases.

## Conclusions

This study investigated real-world treatment outcomes for DME in IVF. In this real-world study, IVF showed good short-term outcomes for DME. IVF may be effective for treating DME in clinical practice.

## Supporting information

S1 Data Raw data of naïve patients. (XLSX)

S2 Data Raw data of switched patients. (XLSX)
